# PRD-Containing Virulence Regulators (PCVRs) in Pathogenic Bacteria

**DOI:** 10.3389/fcimb.2021.772874

**Published:** 2021-10-19

**Authors:** Joseph S. Rom, Meaghan T. Hart, Kevin S. McIver

**Affiliations:** ^1^ Cell Biology & Molecular Genetics, University of Maryland, College Park, MD, United States; ^2^ Maryland Pathogen Research Institute, University of Maryland, College Park, MD, United States

**Keywords:** PCVR, PTS phosphorylation, AtxA, Mga, Mga*Spn*, MafR, nucleoid associated protein

## Abstract

Bacterial pathogens rely on a complex network of regulatory proteins to adapt to hostile and nutrient-limiting host environments. The phosphoenolpyruvate phosphotransferase system (PTS) is a conserved pathway in bacteria that couples transport of sugars with phosphorylation to monitor host carbohydrate availability. A family of structurally homologous PTS-regulatory-domain-containing virulence regulators (PCVRs) has been recognized in divergent bacterial pathogens, including *Streptococcus pyogenes* Mga and *Bacillus anthracis* AtxA. These paradigm PCVRs undergo phosphorylation, potentially *via* the PTS, which impacts their dimerization and their activity. Recent work with predicted PCVRs from *Streptococcus pneumoniae* (Mga*Spn*) and *Enterococcus faecalis* (MafR) suggest they interact with DNA like nucleoid-associating proteins. Yet, Mga binds to promoter sequences as a homo-dimeric transcription factor, suggesting a bi-modal interaction with DNA. High-resolution crystal structures of 3 PCVRs have validated the domain structure, but also raised additional questions such as how ubiquitous are PCVRs, is PTS-mediated histidine phosphorylation *via* potential PCVRs widespread, do specific sugars signal through PCVRs, and do PCVRs interact with DNA both as transcription factors and nucleoid-associating proteins? Here, we will review known and putative PCVRs based on key domain and functional characteristics and consider their roles as both transcription factors and possibly chromatin-structuring proteins.

## Introduction

**Figure 1:**
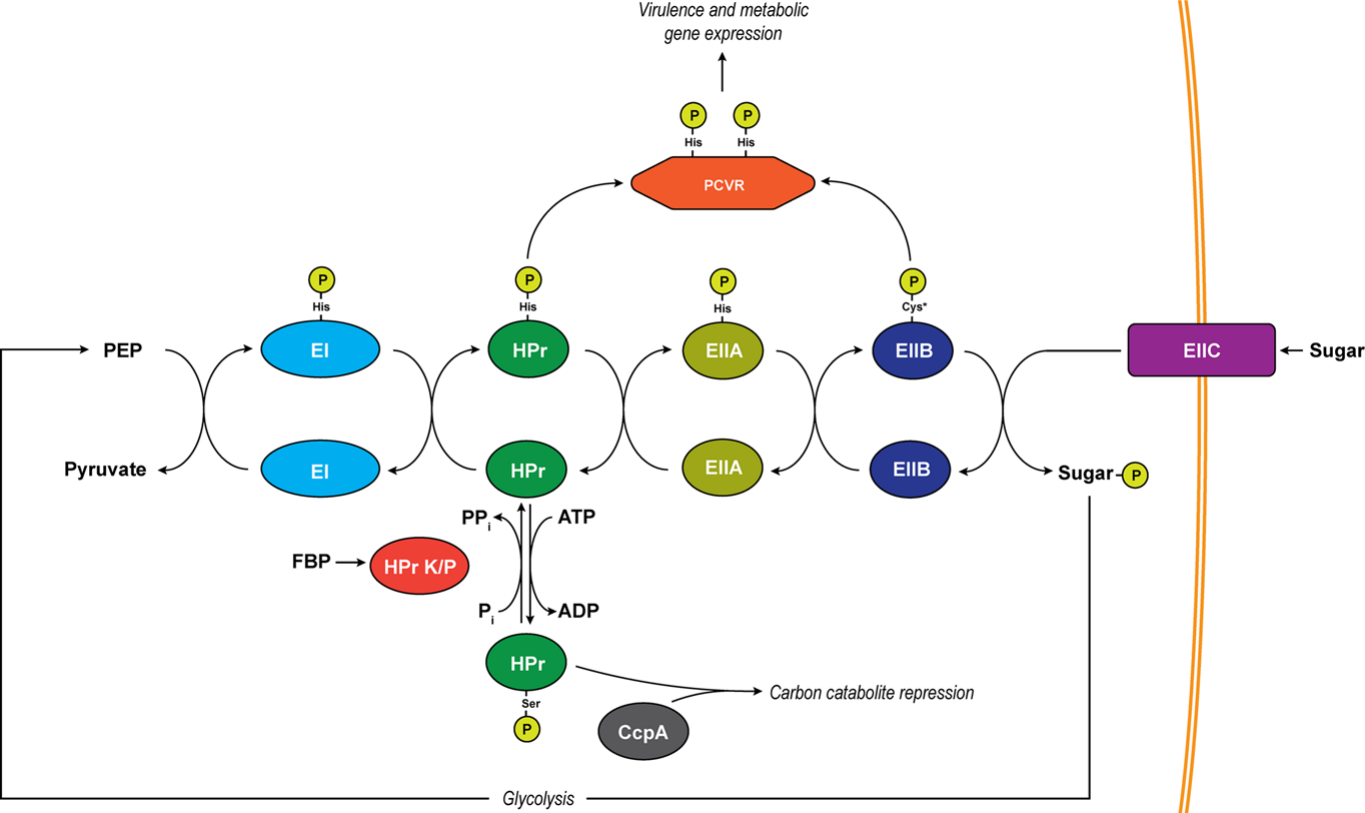
The PTS system. Depicted is a generalized schematic of the PTS that exists in Firmicutes. Phosphates are transferred across conserved histidine residues on EI, HPr, and EIIA. A conserved cysteine serves as the site of phosphorylation on EIIB (asterisk indicates an exception to this in mannose-specific EIIBs which are phosphorylated on a histidine). Major regulatory pathways diverging from the pathway include PCVRs which are putatively controlled through phosphorylation on PRD histidine residues by HPr and/or EIIB proteins as well as carbon catabolite repression through phospho-Ser-HPr. The transition of unphosphorylated HPr to phospho-Ser-HPr is initiated by abundance of ATP as well as the activation of HPr K/P by fructose-1,6-bisphosphate (FBP). HPr K/P can also transfer the phosphate from phospho-Ser-HPr by moving it from HPr to a substrate phosphate (P_i_), resulting in the formation of pyrophosphate (PP_i_).

Nutrient acquisition is a major challenge for bacterial pathogens during infection. Energy-rich carbon resources are in high demand for both the invading pathogen as well as the host. As a result, these resources are limited by the host. In order to overcome these challenges, bacterial pathogens have evolved to produce virulence factors (VFs) as well as nutrient-sensing regulatory proteins to facilitate their expression. In Firmicutes, the phosphoenolpyruvate (PEP) phosphotransferase system (PTS) facilitates the uptake of both preferred (glucose) as well as alternative carbon nutrients by coupling their import and phosphorylation through sugar-specific membrane channels (**Figure 1**) (Deutscher et al., 2014). PEP, a product of glycolysis, provides the energy needed for sugar uptake by donating a phosphate to a series of proteins (EI, to HPr, to a sugar-specific EIIA and EIIB) and finally onto the sugar itself, which is transported into the cell through a cognate EIIC membrane channel (Deutscher et al., 2014).

The availability of nutrients affects the rate of phosphate transfer through the PTS pathway, and as a result, the metabolic state of the cell is regulated based on these nutrient signals. In Firmicutes, one of the major ways bacteria facilitate metabolic activity is through control of carbon catabolite repression (CCR) (Deutscher, 2008). CCR shunts off of the PTS when heat-stable protein (HPr) is phosphorylated by HPr kinase/phosphorylase (HPr K/P), which is activated when preferred sugars, like glucose, yield high levels of ATP (Singh et al., 2008). This activation leads to phosphorylation of HPr at a serine-46 and allows phospho-Ser46-HPr to bind to the carbon catabolite protein A (CcpA), leading to the repressed expression of alternative carbon metabolism genes (Henkin et al., 1991; Deutscher, 2008). Unlike ATP, PEP is in low abundance when readily metabolized sugars are present due to the transfer of phosphate through the PTS (Hogema et al., 1998; Bettenbrock et al., 2007), which occurs *via* the following steps: enzyme I (EI) receives phosphates from PEP on the histidine-191 residue and relays it to histidine-15 on HPr. From phospho-His15-HPr, the phosphate can be transferred to a sugar-specific enzyme IIA (EIIA) protein (Deutscher et al., 2014). An inevitable depletion of preferred sugars results in a decrease in cytosolic ATP, which reduces HPr K/P activity, yielding a greater phospho-His15-HPr/phospho-Ser46-HPr ratio, releasing CcpA-mediated repression of alternative carbon utilization genes, and allowing the bacteria to utilize alternative sources of carbon for energy. Eventually, alternative carbon sources may become scarce for an invading pathogen, creating a decrease in PTS-sugar influx and a buildup of phosphorylated PTS intermediates (EI, HPr, and EIIA/B). These alterations in phosphotransfer modulate the phosphorylation of classical PTS-regulatory domain (PRD)-containing transcription factors and antiterminators. See Görke and Stülke for a comprehensive review of CCR and the PTS (Gorke and Stulke, 2008). This review focuses on a new category of activators termed PRD-containing virulence regulators (PCVRs) that regulate genes needed for a pathogen to adapt to its new microenvironment and cause disease.

In addition to their role in modulating virulence gene expression, PCVRs play an important role in nutrient utilization (Uchida et al., 1993; Hoffmaster and Koehler, 1997; Ribardo and McIver, 2006). They share important structural and functional similarities with classical PRD-containing antiterminators (e.g., LicT) and transcriptional activators (e.g., LevR and MtlR), most notably by possessing specific histidine residues at sites of phosphorylation within PRDs that modulate their activity (Schnetz et al., 1996; Deutscher et al., 2014). PCVRs appear to function like transcriptional regulators, binding DNA at specific intergenic regions and promoting or repressing gene expression accordingly (Chen et al., 1993; Uchida et al., 1993; McIver et al., 1995; McIver et al., 1999; Hemsley et al., 2003; Almengor and McIver, 2004; Almengor et al., 2006; Ruiz-Cruz et al., 2015; McCall et al., 2019). Nucleotide sequence specificity may be less important than the intrinsic curvature and adjacent bendability of the DNA in mediating these interactions (Hadjifrangiskou and Koehler, 2008; Solano-Collado et al., 2016). Interestingly, PCVR transcriptional activity appears to be directly proportional to their multimeric state at specific *cis*-regulatory elements (Hammerstrom et al., 2011; Solano-Collado et al., 2013; Ruiz-Cruz et al., 2015), a trait shared with nucleoid-associating proteins (NAPs). These observations have raised the possibility that PCVRs are descendants of prototypical PRD-containing transcriptional activators and have evolved functional characteristics of genome-modeling architectural proteins. In this review, we will use the two-best characterized PVCRs, the *Bacillus anthracis* AtxA and the *Streptococcus pyogenes* Mga to establish a definition of a PCVR and how these regulators may differ from classical PRD-containing regulatory proteins. Furthermore, we will discuss cutting-edge experimental techniques that could provide novel insights into understanding how PCVRs function as PTS-responsive elements that link metabolism and virulence in Gram-positive pathogens.

## Prototypic PCVRs in Pathogenic Bacteria

### PTS Sugar Uptake and Regulation

PCVRs share the greatest organizational similarity with the *B. subtilis* mannitol operon transcriptional activator, MtlR (Deutscher et al., 2014), which consists of two N-terminal nucleic acid-binding domains, two centralized PRD-domains with conserved phosphorylated histidine residues, and a C-terminal EIIB-like domain (**Figure 1**). In MtlR, both DNA-binding domains have a helix-turn-helix secondary structure (pfam HTH_11) (Pabo and Sauer, 1984; Brennan and Matthews, 1989). The second DNA-binding domain also contains an HTH-motif, sharing sequence similarity to the Mga HTH-domain (HTH^Mga^) that will be discussed later. The internal PRD domains (PRD-1 and PRD-2, respectively) contain histidine residues (230 and 289 for PRD-1 and 342 and 399 for PRD-2) which in MtlR, are spatially conserved and subject to PTS-phosphorylation (Deutscher et al., 2006). The C-terminal region of MtlR contains two EII-like binding domains with the proximal galactitol-specific EIIB-like domain (EIIB^Gat^) containing a conserved cysteine at position 419, which is immediately followed by a distal EIIA-like binding domain which contains a conserved histidine at position 599 (Deutscher et al., 2014). Unlike MtlR, PCVRs always appear truncated, lacking the most C-terminal EIIA-like domain. Additionally, the location of histidine residues in PCVR PRDs are not spatially conserved, although in some instances, they are still subject to phosphorylation *in vitro* (Tsvetanova et al., 2007; Hondorp et al., 2013).

In addition to the similarities in domain organization defined by secondary structure, protein data bank (PDB) crystal structures of AtxA (PDB 4R6I) as well as two homologs, *Streptococcus pneumoniae* Mga*Spn* (PDB 5WAY) and *Enterococcus faecalis* MafR/EF3013 (PDB 3SQN), show similarities in tertiary structure to established PRD-containing regulators (Osipiuk et al., 2011; Hammerstrom et al., 2015). This raises the question that PCVRs may share a common ancestral protein with a classical PRD-containing activator, perhaps even MtlR itself. Before discussing this topic further, we want to describe the structural and functional similarities that are conserved amongst PCVRs, starting with the two-best characterized PCVRs, the *Bacillus anthracis* AtxA and the *Streptococcus pyogenes* Mga.

### AtxA

The anthrax toxin activator (AtxA) is a 53-kDa, DNA-binding protein that is encoded by *atxA* located between the *cya* and *pagA* genes on the *Bacillus anthracis* pXO1 plasmid (Uchida et al., 1993; McCall et al., 2019). AtxA was first characterized as a major transcriptional activator of the pXO2 plasmid-encoded capsule genes (*capBCADE*) as well as the tripartite toxin genes encoding protective antigen (*pagA*), lethal factor (*lef*), and edema factor (*cya*) located on the pXO1 plasmid (Leppla, 1982; Green et al., 1985; Uchida et al., 1985; Robertson and Leppla, 1986; Uchida et al., 1987; Makino et al., 1988; Tippetts and Robertson, 1988; Makino et al., 1989). AtxA has positive and negative effects to varying degrees on different genes through indirect mechanisms of regulation (Hoffmaster and Koehler, 1997; Drysdale et al., 2004; Mignot et al., 2004; Corsi et al., 2021). Virulence of *B. anthracis* appears to be modulated directly through AtxA, as mutation of *atxA* resulted in reduced transcription of *pagA*, *lef*, and *cya* and correlated with attenuated virulence in a mouse model of infection (Dai et al., 1995). *Bacillus cereus* strain G9241, which causes anthrax-like disease, possesses two alleles of *atxA* (*atxA1* and *atxA2*). Loss of both AtxA orthologues (AtxA1 and AtxA2) attenuated virulence in a capsule-dependent manner, thus highlighting AtxA as a master regulator which enables the virulence of pathogenic *Bacilli* (Scarff et al., 2016).

### Mga

The multiple gene activator of the Group *A* streptococcus (Mga) is a 62-kDa DNA-binding protein and a major stand-alone virulence regulator conserved in all strains of *S. pyogenes* (Podbielski, 1992; Kreikemeyer et al., 2003; Bessen et al., 2005). There are two divergent isotypes, *mga-1* and *mga-2*, that are associated with strains causing throat infections and skin infections, respectively (Bessen et al., 2005). The *mga* gene is encoded just upstream of the *emm* gene, which encodes the major *S. pyogenes* surface M-protein or a related *emm*-like gene (Spanier et al., 1984; Caparon and Scott, 1987), and can positively control its own expression (Geist et al., 1993; Podbielski et al., 1995; McIver et al., 1999). It enhances the expression of different virulence genes, including those encoding the fibronectin-binding serum opacity factor (*sof*) and the collagen-like protein A (*sclA*) (Caparon and Scott, 1987; Chen et al., 1993; McLandsborough and Cleary, 1995; Lukomski et al., 2000b; Rasmussen et al., 2000) and a cluster of virulence factor-encoding genes (Mga locus) that includes *scpA* (encoding the C5a peptidase), *sic* (encoding the secreted inhibitor of complement), and *fba* (encoding the fibronectin-binding surface adhesin) (Haanes et al., 1992; Podbielski, 1993; Hollingshead et al., 1994). The activity of Mga is growth phase-dependent, being most active during exponential phase growth leading to enhancement of adhesion and immune evasion (McIver and Scott, 1997), while modulating metabolism during early infection (Ribardo and McIver, 2006). Numerous studies have shown that an intact Mga operon is required for *S. pyogenes* to cause disease, making it an attractive therapeutic target (Kihlberg et al., 1995; Schmidt et al., 1996; Courtney et al., 1999; Lukomski et al., 2000a).

### PCVR Structural Characteristics

One potential problem with the hypothesis that PCVRs are descended from a common ancestral PRD-containing protein lies in that they generally lack sequence similarity. Nevertheless, the more obvious structural similarities of PCVRs, specifically at the secondary and tertiary levels of order, suggest that PCVRs are indeed orthologues and a product of either divergent or convergent evolution. Despite having a crystal structure available for AtxA (PDB 4R6I), Mga has proven exceedingly difficult to express to levels that would allow it to be purified for crystallography. To determine the homology of PCVR functional domains, a Phyre2 analysis was used comparing user input amino-acid sequences to those of known three-dimensional structures (Kelley et al., 2015). AtxA was used as a template for these comparisons, allowing other PCVR peptide sequences to be aligned to the secondary structural “landmarks” defined in AtxA.

**Figure 2:**
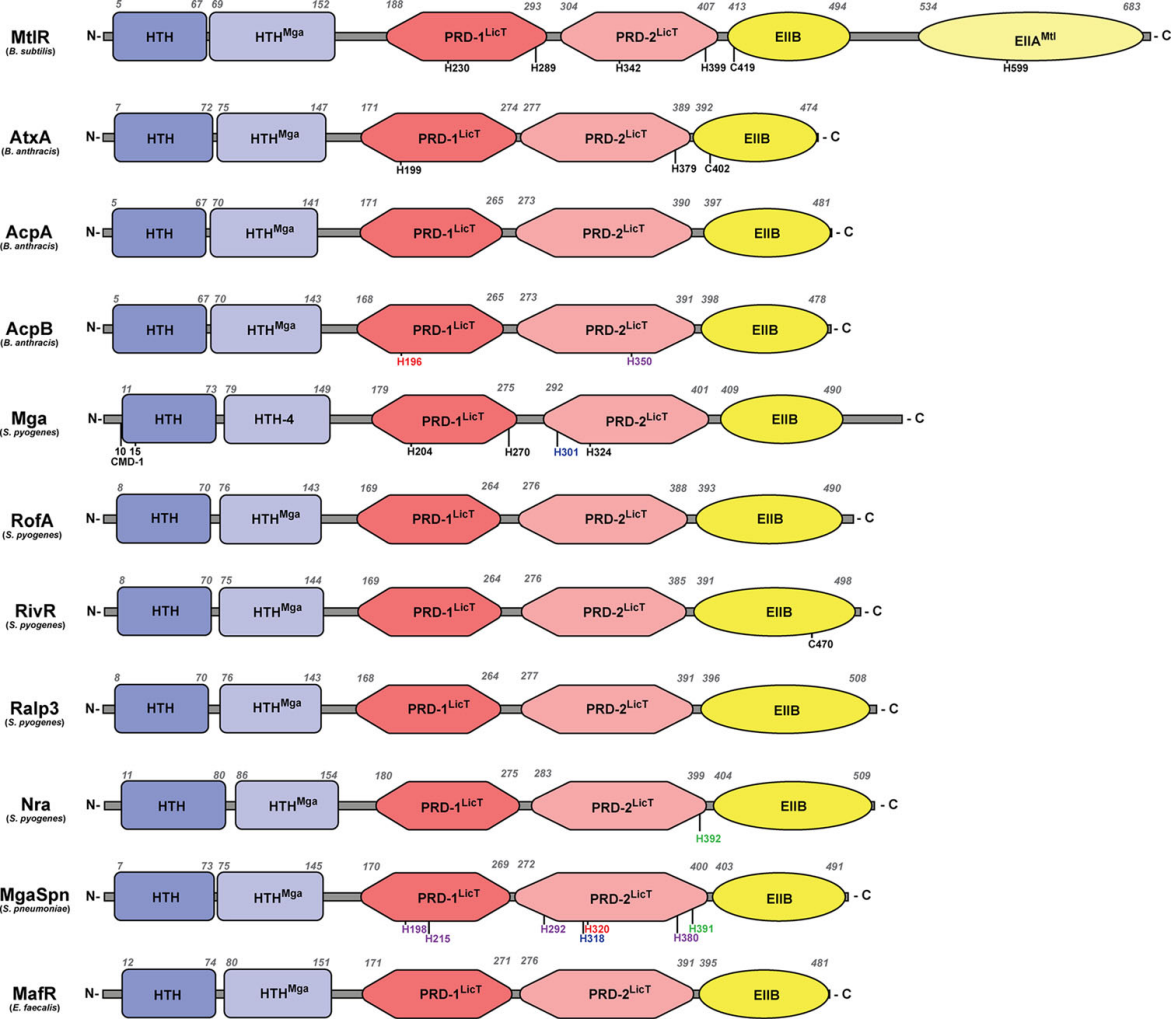
Structural domains of PCVRs. Structural domains of each PCVR, with MtlR included for comparison, include two putative DNA-binding domains at the N-terminus, two central PRDs, and one EIIB-like domain that is structurally similar to the EIIBs specific for galactitol, mannose, and lactose. Italicized numbers above each peptide indicate amino acid residues as landmarks starting from the N-terminus. Black residues are sites of phosphorylation, with the exception of C402 and C470 which are implicated in dimerization in AtxA and RivR, respectively. Green residues align with AtxA residues that undergo phosphorylation. Red residues align with Mga residues that undergo phosphorylation. Blue residues align with MtlR residues that undergo phosphorylation. Purple residues align with AtxA and/or Mga but have no known ability to undergo phosphorylation. Conserved Mga domain (CMD-1).

As previously mentioned, both AtxA and Mga share predicted domain organization with MtlR, including two N-terminal DNA-binding domains sharing HTH-motif structural homology (**Figure 2**) (McIver and Myles, 2002; Tsvetanova et al., 2007). The first HTH domain has homology to the ubiquitous HTH_11 Pfam category found amongst many DNA-binding proteins (Wilson et al., 1992), while the second HTH domain shares similar primary structure originally identified as HTH-4 in Mga and given the Pfam designation HTH^Mga^ (Podbielski et al., 1995; McIver and Myles, 2002). McIver and Myles used mutagenesis to show that the HTH-3 domain (HTH_11) was partially required for DNA-binding whereas the HTH-4 domain (HTH^Mga^) was absolutely essential for binding (McIver and Myles, 2002). Mga also contains six conserved N-terminal amino acids (QQWREL) known as the conserved Mga domain (CMD-1) that is found in Mga as well as some *S. dysgalactiae* Mga orthologues (Vahling and McIver, 2006). In *S. pyogenes*, the Mga CMD-1 is involved in promotor binding in both *emm* expression and *mga* auto-regulation (Vahling and McIver, 2006). However, CMD-1 is not conserved in the orthologues Mga*Spn* and MafR, although each protein shares a conserved arginine (R) residue and in the case of Mga*Spn*, a conserved leucine (L) residue as well.

As the PCVR name implies, AtxA and Mga contain two central LicT-like PRDs as well as a C-terminal EIIB-like domain that shares sequence similarity with a region found in the galactitol-specific EIIB (EIIB^Gat^) in *E. coli* (Volpon et al., 2006; Tsvetanova et al., 2007). Within their PRDs, AtxA and Mga contain histidine residues that are believed to undergo phosphorylation in response to PTS signals, with H199 (PRD-1) and H397 (PRD-2) in AtxA and H204/H270 (PRD-1) and H324 (PRD-2) in Mga (Tsvetanova et al., 2007; Hondorp et al., 2013). Unlike AtxA, the histidine residues of Mga closely align with the PRD-contained histidine residues of LicT, LevR, and MtlR (Deutscher et al., 2014).

### PCVR Modulation by the PTS

**Figure 3:**
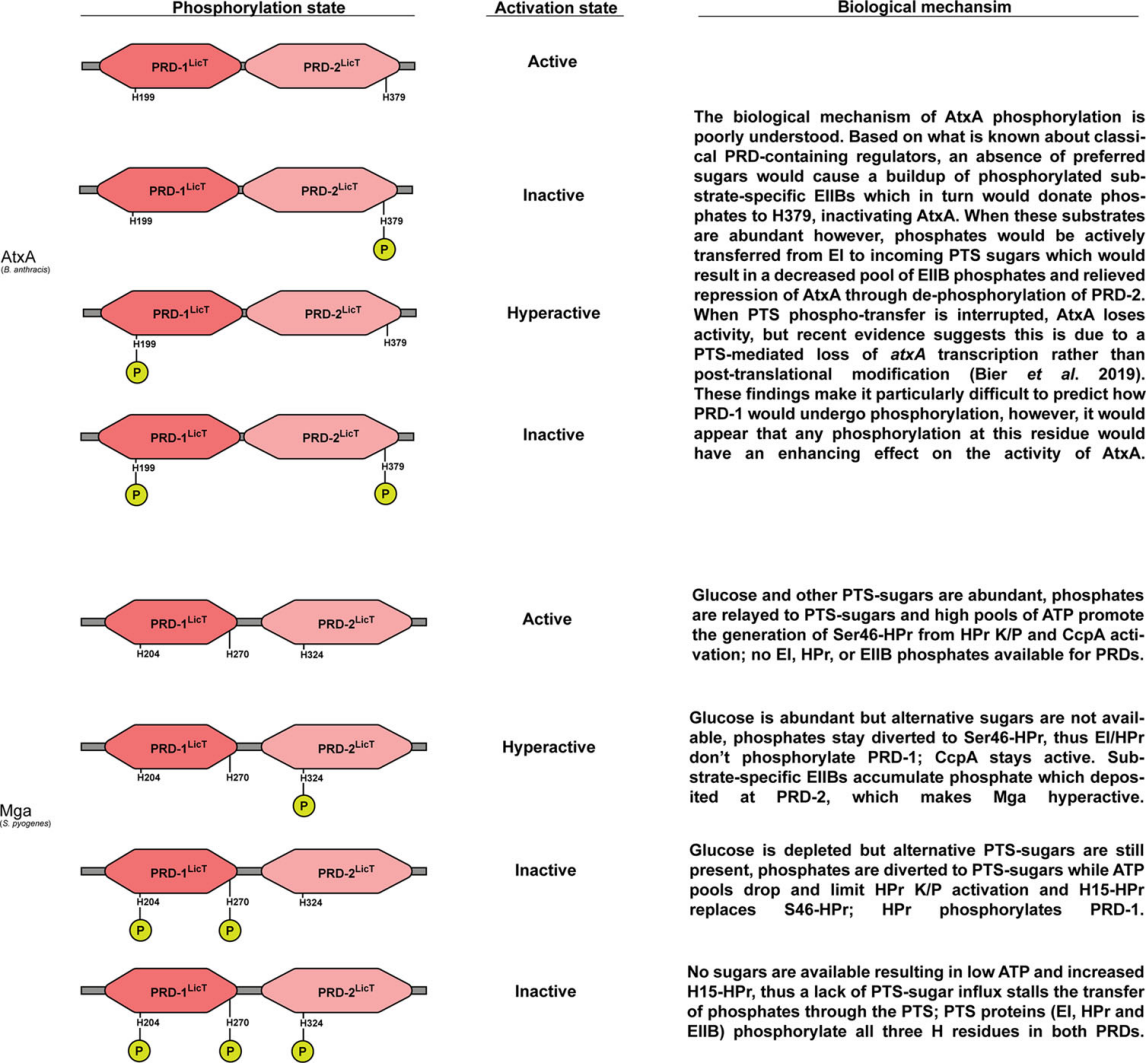
Functional status and hypothesized biochemical inputs that regulate AtxA and Mga PRDs. Left: Predicted phosphorylation patterns for PRDs contained within AtxA and Mga. Center: Resulting activity levels given respective phosphorylation patterns; note that phosphorylation at the PRD2 has a dominant role on PCVR activation in AtxA whereas phosphorylation in PRD1 dominates the activation state of Mga. Right: Predicted biological inputs resulting in respective phosphorylation and PCVR activation states.

The involvement of the PTS in regulating AtxA and Mga through post-translational modifications (PTMs) is poorly understood (**Figure 3**). Classical PRD-containing regulators can be phosphorylated by EI, HPr and substrate-specific EIIB proteins in both PRDs and in the EII-like domains under a variety of conditions (Martin-Verstraete et al., 1998; Lindner et al., 1999; Tortosa et al., 2001; Xue and Miller, 2007; Joyet et al., 2010; Joyet et al., 2013). Deutscher et al. provides a good review of how PTS proteins interact and phosphorylate different regulatory domains in PRD-containing regulators (Deutscher et al., 2014). The canonical mechanism of PTS regulation involves EI/HPr phosphorylating one PRD-containing regulatory element to activate the regulator, while the EIIB protein phosphorylates another PRD, inactivating the regulator.

There is little direct evidence that PTS proteins (EI, HPr, EIIB) interact with AtxA. However, a series of experiments using site-directed mutagenesis to induce phospho-mimetic (H to D) and phospho-ablative (H to A) amino acid substitutions in AtxA revealed that H199D (in the PRD-1) activated AtxA while H379D (in the PRD-2) had an inhibitory effect (Tsvetanova et al., 2007; Hammerstrom et al., 2015; McCall et al., 2019). Specifically, active AtxA upregulated the expression of *pagA* and *lef*, formed dimers in solution, and directly bound to DNA. Interestingly, the H379D substitution had a dominant effect on AtxA activity in that it suppressed *lef* expression as well as the production of capsule irrespective of the amino acid residue present at position 199 (Hammerstrom et al., 2015). In addition to these intriguing findings, Tsvetanova and colleagues found that AtxA could be phosphorylated and immunoprecipitated from *B. anthracis* cell lysates, but when phospho-ablative substitutions were made at both PRD histidine residues (H199A and H379A), AtxA could no longer be immunoprecipitated in a phosphorylated form (Tsvetanova et al., 2007). Taken together, these reports suggest that AtxA activity may be directly linked to the PTS and phosphorylation. However, a recent report by Bier and colleagues has challenged the hypothesis that AtxA is directly phosphorylated by PTS proteins (Bier et al., 2020). They found that EI and HPr did not directly phosphorylate AtxA *in vitro*, but instead, an intermediate regulatory protein is likely phosphorylated by the PTS which in turn modulates the expression of *atxA*. This was based on a series of experiments whereby isopropylthio-β-galactoside (IPTG)-inducible *atxA* expression (PTS-independent) had no altered ability to express the *B. anthracis* toxin *lef*, regardless of the presence or absence of an intact PTS. In contrast, *atxA* under the control of its native promotor was unable to induce *lef* expression in a PTS-null (mutated for *ptsH* and *ptsI* that encode HPr and EI, respectively) genetic background. Additionally, *atxA* expression was significantly decreased in a *ptsHI* double mutant. EIIB was not investigated for *in vitro* phosphorylation of AtxA, thus its role in this process still needs to be explored.

Although there is no evidence of direct PTS phosphorylation of Mga *in vivo*, Valdes and colleagues demonstrated that Mga does undergo variable phosphorylation *in vivo* (Valdes et al., 2018). Unlike AtxA, Hondorp et al. provided clear evidence that Mga from serotype M4 (*mga-2*) and M1 (*mga-1*) could be phosphorylated by the PTS *in vitro* using purified PEP, EI, and HPr (Hondorp et al., 2013; Bier et al., 2020). Combinations of phospho-mimetic and -ablative mutations targeting the histidine residue codons in *mga* yielded a collection of mutants with differential Mga activity (**Figure 3**). In contrast to MtlR and AtxA, phospho-mimetic mutations in PRD-1 of Mga (H204D and H270D) led to the protein being inactivated, and this phenotype was found to be dominant over a phospho-mimetic mutation in PRD-2 (H324D) that activated the protein (Joyet et al., 2010; Hondorp et al., 2013; Sanson et al., 2015). These studies provide evidence that PCVRs like AtxA and Mga can be modified by the PTS through phosphorylation events within PRDs, but unlike well-studied PRD-containing regulator MtlR, the exact mechanism by which the PTS controls these PCVRs may not be conserved. Further studies will be required to determine if *in vitro* phosphorylation events are representative of PTMs that occur *in vivo* through the PTS.

### PCVR Multimerization

Like MtlR, both AtxA and Mga share a C-terminal EIIB^Gat^-like domain (Tsvetanova et al., 2007; Hondorp et al., 2012). There is accumulating evidence that this region is required for the dimerization and the subsequent transcriptional regulatory activity of each protein. Specifically, Hammerstrom and colleagues found that mutating the codon for the cysteine (C402) in the EIIB^Gat^ domain of AtxA prevented it from forming homo-dimers using a co-immunoprecipitation approach (Hammerstrom et al., 2011). Subsequent studies found that an H199A substitution impaired the ability of AtxA to bind DNA, (McCall et al., 2019) whereas both H379D and H379E substitutions reduced its capacity to form homodimers, presumably through loss of EIIB interactions resulting from changes in protein conformation (Hammerstrom et al., 2015). In Mga, multimerization is readily observed when the solution undergoes changes in osmolality and pH. Divalent cations like Ni^2+^ and Zn^2+^ cause Mga to form large aggregates in solution that have hampered the ability to purify the protein (Hondorp et al., 2012). Yet, Hondorp and colleagues found that deleting the entire EIIB^Gat^-like domain of Mga eliminated Mga homo-multimerization as well as transcriptional activity. They later found that phospho-mimetic double mutations (H204D/H270D) in the PRD-1 of both isotypes of Mga prevented multimerization (Hondorp et al., 2013). Interestingly, Mga was still able to bind DNA as a monomer, however, the transcriptional activation by non-multimerizing Mga was impaired, implying that multimerization is required for the full regulatory potential of Mga. Thus, PTS phosphorylation likely impacts the activity of both AtxA and Mga by altering their ability to form multimers, yet the exact pathway by which these PTS signals are transduced is not fully understood.

## Novel PCVRs in Gram-Positive Pathogens

Based on homology with the MtlR, Mga, and AtxA, additional PCVRs can be found in the genomes of diverse species of Gram-positive pathogenic bacteria. In this section, we will describe more recently discovered candidates and discuss the basis for their classification as PCVRs.

### AtxA Paralogs

*B. anthracis* contains two AtxA paralog activators of capsule synthesis (AcpA and AcpB), both of which are encoded on the 96-kb pXO2 plasmid and have been implicated in regulating virulence (Vietri et al., 1995; Drysdale et al., 2004; Raynor et al., 2018). The role of AcpA and AcpB in modulating virulence is thought to be primarily attributed to an AtxA-induced expression of the two proteins, which in turn control the expression of capsule through direct *capBCADE* promotor binding (Bourgogne et al., 2003; Drysdale et al., 2004). Two reports show that both *acpA* and *acpB* impact virulence *in vivo*, however each report drew opposing conclusions as to which gene is more important and whether the genes are synergistic or redundant (Drysdale et al., 2005; Sittner et al., 2021). It should be noted that the authors used dissimilar animal models for their studies.

The genes encoding AcpA and AcpB are located in close proximity to one another (Vietri et al., 1995; Drysdale et al., 2004), and have been described as PCVRs based on their sequence similarity with AtxA and their function (Raynor et al., 2018). Both AcpA and AcpB contain two N-terminal HTH putative DNA-binding domains as well as two core LicT-like PRDs and one C-terminal EIIB^Gat^-like domain (Raynor et al., 2018) (**Figure 2**). Both AcpA and AcpB contain numerous histidine residues in their predicted PRDs, yet AcpA lacks any overlap with PRD histidine residues in AtxA, Mga, and MtlR. On the other hand, AcpB H350 aligns with a histidine residue in both AtxA and Mga. Although there are no reports of these histidine residues affecting protein activity, the H196 of AcpB aligns with the H230 of MtlR and H204 of Mga, both of which are important in the phosphorylation and activity of each respective protein (Joyet et al., 2010; Hondorp et al., 2013; Heravi and Altenbuchner, 2014).

Like AtxA, the activities of AcpA and AcpB are dependent on multimerization. Co-immunoprecipitation experiments revealed that epitope-tagged AcpA and AcpB form homodimers and that AcpA may also form heterodimers with AtxA (Raynor et al., 2018). Mutation of the C-terminal EIIB-like domains in either AcpA or AcpB relieved this association and also led to a loss of *capB* expression activity, which is consistent with previous studies showing that homo-, and in this case, hetero-multimerization directly affect the transcriptional activity of AcpA and AcpB.

### Mga Paralogs

*S. pyogenes* contains four Mga paralogs, RofA, Nra, Ralp3 and RivR that, given their structural similarities to RofA and one another, comprise a family termed the RofA-like proteins (RALPs) (Granok et al., 2000). Their presence and combination vary with serotype and contribute to a heterogenous pattern of gene expression (Podbielski et al., 1999; Kreikemeyer et al., 2002; Kreikemeyer et al., 2007; Buckley et al., 2020). Each of these paralogs have been implicated in regulating known virulence factors and adhesins that contribute to GAS fitness and viability *in vitro* (Molinari et al., 2001; Kreikemeyer et al., 2002; Kwinn et al., 2007; Siemens et al., 2012; Trevino et al., 2013). Additionally, deletion of *ralp3* and *rivR* have been shown to attenuate virulence in murine models of GAS infection (Kwinn et al., 2007; Trevino et al., 2013). Interestingly, the native Ralp3 coding sequence in the M1T1 strain, studied by Kwinn and colleagues, contains a nonsense mutation about halfway through the gene, suggesting this version of Ralp3 is truncated and would lack the second hypothesized PRD domain and the EIIB domain. Despite this truncation, their results suggest that Ralp3 still maintains a role in regulating the virulence of this strain.

Like Mga, secondary structure predictions by Phyre2 suggest the RALPs closely resemble AtxA. Each RALP has two N-terminal HTH domains, two central PRDs with several histidine residues, and one C-terminal EIIB^Gat^-like domain (Kelley et al., 2015) (**Figure 2**). There is currently no evidence that any of the RALPs are capable of undergoing phosphorylation or if the PTS modulates their activity. However, Phyre2 analysis showed that Nra from the M49 serotype of *S. pyogenes* contains a histidine residue (H392) in its PRD-2 that aligns with the H379 in AtxA, thus opening up the possibility that PTS-mediated phosphorylation occurs in RALPs (Kelley et al., 2015).

To the best of our knowledge, the only RALP in which multimerization has been studied is RivR, which was assessed *in vivo* by cross-linking (Ramalinga et al., 2016). The resulting banding patterns indicated that RivR formed both dimers and multimers in the cell. Substitution of cysteine-470, located in the EIIB^Gat^-like domain, to serine (C470S) completely ablated the ability of RivR to form dimers, and inhibited RivR from regulating its target genes. This suggests the EIIB^Gat^-like domain is primarily responsible for multimerization, which is consistent with AtxA and Mga (Hammerstrom et al., 2011; Hondorp et al., 2012). Direct DNA binding has been demonstrated *in vitro* for RofA, which binds two sites in the intergenic region between the RofA-regulated genes *rofA* and *prtF* of a serotype M6 strain (Granok et al., 2000). Analysis of the two RofA binding sites within the intergenic region identified a consensus binding sequence, however, this sequence is not found upstream of any other RofA-regulated genes (Beckert et al., 2001). The absence of RofA binding sites could suggest indirect regulation by RofA or that RofA can bind DNA both in a sequence-dependent and -independent manner. Analysis of the region upstream of RivR-regulated genes also failed to identify a consensus sequence (Trevino et al., 2013), suggesting sequence-independent binding or indirect regulation.

### Mga*Spn*

Pioneering work found that the *Streptococcus pneumoniae* isolate TIGR4 contains a 58-kDa protein that repressed the *rlrA* pathogenicity locus (Hava et al., 2003; Hemsley et al., 2003). This gene, originally called *mgrA* (SP1800), shares a high degree of amino acid sequence similarity to *S. pyogenes* Mga (Hemsley et al., 2003; Solano-Collado et al., 2012). Because of its similarities, MgrA was renamed Mga of *Streptococcus pneumoniae* (Mga*Spn*). Like Mga, Mga*Spn* plays an important role in early infection by modulating the production of bacterial adhesins encoded within the *rlrA* pathogenicity islet that provide *S. pneumoniae* the capacity to colonize the nasopharynx and progress to pneumonia (Hemsley et al., 2003). Interestingly *rlrA* positively regulates the other genes of the *rlrA* islet and is homologous to RofA and Nra of *S. pyogenes* (Hava et al., 2003). Furthermore, the *rlrA* islet has significant homology to the type-1 fibronectin-collagen-T-antigen (FCT) encoding region of *S. pyogenes* (Nakata and Kreikemeyer, 2021), suggesting that *rlrA* and the type-1 FCT loci are derived from an ancestral genetic element.

Mga*Spn* has been successfully crystallized (PDB 5WAY) and PDB prediction software shows that it forms a dimer with the C-terminal regions in close proximity to each other with the N-terminal regions facing out. Phyre2 alignments with AtxA show that Mga*Spn* contains secondary structures that are consistent with two N-terminal HTH-domains, two central PRDs, and a C-terminal EIIb^Gat^-like domain (**Figure 2**) (Kelley et al., 2015). TIGR4 Mga*Spn* PRD residues H215 and H292 align with histidine residues of Mga, and Mga*Spn* H198 and H380 align with histidine residues of AtxA; however, none of these are established sites of PTS-phosphorylation in Mga or AtxA. Interestingly, Mga*Spn* H391, H320, and H318 align with H379, H324, and H342 of AtxA, Mga, and MtlR, respectively, all of which have been shown to be involved in modulating protein activity (Tsvetanova et al., 2007; Hondorp et al., 2013; Joyet et al., 2013). These overlaps in histidine residues would indicate that Mga*Spn* may still retain the capacity to undergo PTS-mediated phosphorylation.

As observed with Mga, purification of Mga*Spn* resulted in the formation of multimers in solution (Solano-Collado et al., 2013). Mga*Spn* also binds conserved regions of DNA, but in a sequence-independent manner and binding is enhanced as multimers aggregate around the site of initial dimerization (Solano-Collado et al., 2012; Solano-Collado et al., 2013). The authors noted that these characteristics are shared with prokaryotic architectural proteins such as nucleoid-associated proteins (NAPs), including HNS, HU, and IHF. To date, the question of whether or not Mga*Spn* can be directly phosphorylated at its PRD domains has not been investigated.

### MafR

*Enterococcus faecalis* encodes a 56-kDa AtxA/Mga homolog MafR (EF3013) that was initially characterized by X-ray crystallization (PDB 3SQN) based solely on homology and biochemical analysis (Osipiuk et al., 2011). Subsequent studies by Ruiz-Cruz and colleagues have found that MafR plays a central role in the regulation of numerous metabolic genes, including both PTS- and non-PTS ABC-transporters, as well as genes involved in calcium and queuosine homeostasis and utilization of glycerol, maltose, and mannitol (Ruiz-Cruz et al., 2015; Ruiz-Cruz et al., 2018; Ruiz-Cruz et al., 2019). To date, there are no reports that suggest a direct link between MafR and the expression of *E. faecalis* virulence genes; however, loss of MafR led to significant attenuation in a murine model of peritonitis, suggesting that the proper regulation of metabolic genes is also crucial in the overall virulence of this opportunistic pathogen (Ruiz-Cruz et al., 2015).

Protein folding prediction based on the crystal structure show folding and dimerization of MafR similar to Mga*Spn*. Phyre2 analysis confirmed that MafR shares the same structural domains as Mga, including two HTH-domains that likely bind DNA (Kelley et al., 2015) (**Figure 2**). Alignments of MafR against MtlR, AtxA, and Mga-1 and -2, revealed no shared histidine residues in the PRDs with any of these other proteins. Like AtxA, Mga, and Mga*Spn*, MafR has the propensity to form multimers and this appears critical for its transcriptional activity (Ruiz-Cruz et al., 2018). Additionally, MafR DNA-binding sites lack conserved sequences, are AT-rich, and contain intrinsic curvature, all three of which are traits shared by NAP binding sites (Ruiz-Cruz et al., 2018; Ruiz-Cruz et al., 2019). The lack of histidine conservation and, more so, the NAP-like behavior of MafR suggest that the protein activation mechanisms have diverged evolutionarily from those of classical PRD-containing regulatory proteins and perhaps have even converged towards those of NAPs. These points will be discussed later on in this review.

## Prevalence of PCVRs in Streptococi

**Figure 4:**
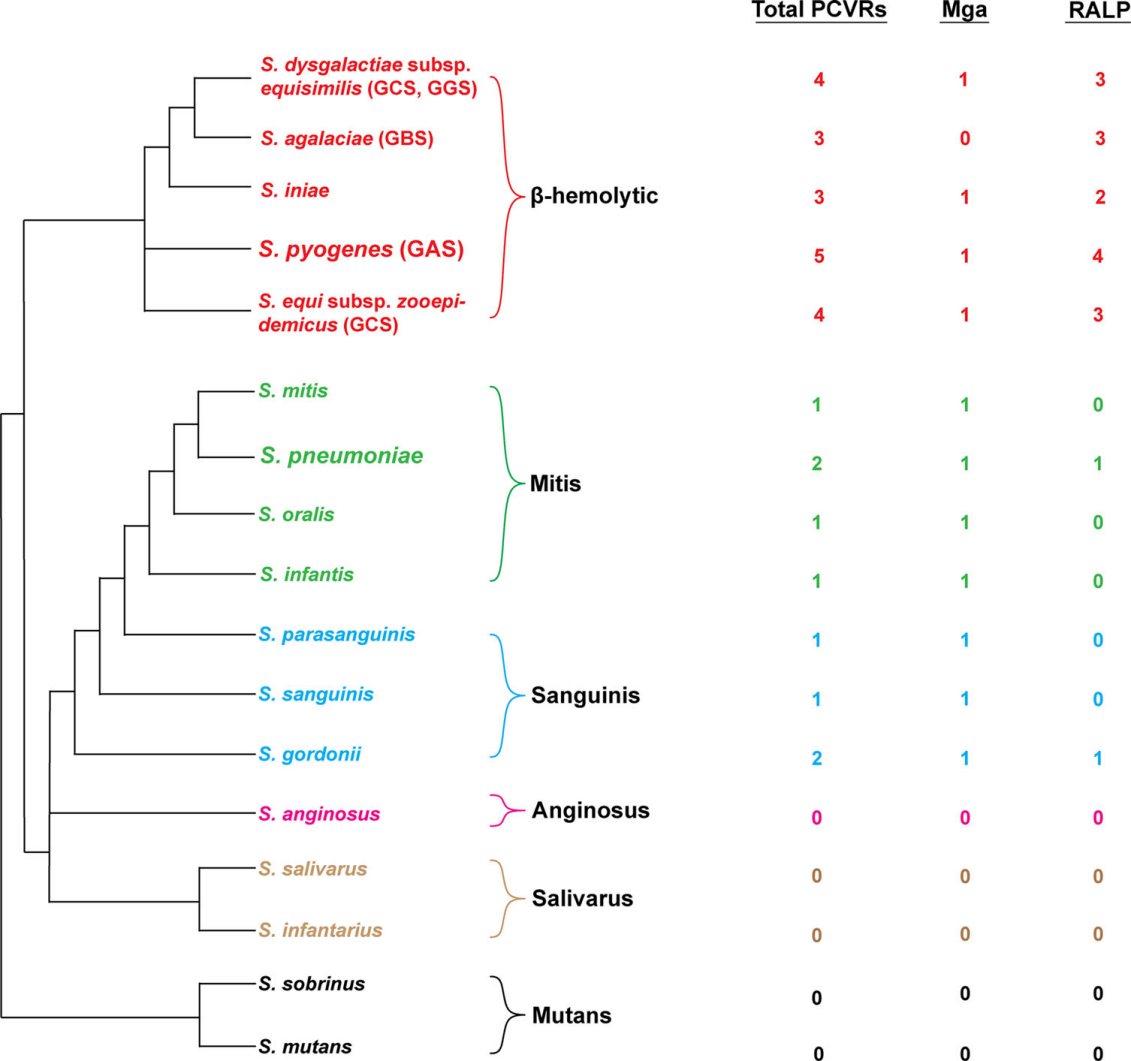
Distribution of PCVRs in streptococcal species. Left: Depicted are the major streptococcal species and clades organized based on their evolutionary divergence. Beta-hemolytic streptococci are located in the top clade in red. Divergent viridans species are organized below. Right: The abundance of Mga/RALP (red) or Mga/RALP-like (green/blue/pink/tan/black) PCVRs are listed for each respective streptococcal species. The Mga/RALP-like PCVRs were identified based on sequence similarities to the M1 *S. pyogenes* strain 5448 using BLAST analysis.

As more AtxA/Mga family proteins are identified, it has become more apparent that PCVRs are a diverse class of regulatory proteins that are widespread in Gram-positive pathogens. In the streptococci, numerous structural homologs of Mga and the RALPs have been previously discovered (Geyer and Schmidt, 2000; Vasi et al., 2000; Hava and Camilli, 2002). The *S. pyogenes* M1T1 isolate 5448 Mga was used to identify homologous proteins in the genomes of multiple pathogenic streptococcal species using BLAST (**Figure 4**). Interestingly, the abundance of Mga and RALP homologs correlated directly with the pathogenic potential of each queried streptococcal species in humans. β-hemolytic species such as Group B (*S. agalactiae*) and Group C streptococci (*S. dysgalactiae* and *S. equi*) exhibit the most homologs (both having one Mga and three RALP homologs) whereas more distantly related viridans streptococci, such as the α-hemolytic *S. pneumoniae* or the γ-hemolytic *S. mutans* having few or no Mga/RALP homologs. These findings support the hypothesis that PCVRs originated from a common ancestral protein that either evolved from a classical PRD-containing regulator or a PRD-containing regulator-like protein that arose convergently. These similarities suggest that the Mga/RALP PCVRs provided a greater fitness advantage to ancestral streptococci that evolved into human pathogens, whereas streptococci that succeeded as commensals did not experience the same selective pressure to retain them.

## Catabolic Signals that Regulate PCVRs

### The Impact of Glucose on PCVRs

Glucose is a preferred carbon source and is also involved in influencing both AtxA- and Mga-regulated virulence genes (Pine and Reeves, 1978; Ribardo and McIver, 2006; Chiang et al., 2011). A recent transcriptomic study in M1T1 *S. pyogenes* grown in either a glucose rich medium (THY) or glucose-limited medium (C media) showed a distinct difference in the Mga regulon under these two different growth conditions (Valdes et al., 2018). Surprisingly, high glucose resulted in phosphorylated Mga, whereas phosphorylated Mga was reduced when grown in low glucose. These results indicate that glucose alters the phosphorylation state and subsequent activity of Mga in *S. pyogenes*. One possible explanation for the observed glucose-induced Mga phosphorylation phenotypes could be that glucose may have a lesser effect on modulating PTS than other undefined sugar sources. Glucose can enter the cell independently of the PTS (Fiegler et al., 1999) and Sundar and colleagues showed that glucose transport in *S. pyogenes* is primarily through the non-PTS GlcU transporter and converted to glucose-6-phosphate by the NagC glucose kinase (Sundar et al., 2018). Nevertheless, results from Hondorp et al., 2013 and Valdes et al., 2018 suggest that Mga activity is a direct consequence of glucose availability, which in turn alters the availability of phosphates that can be transferred from PTS proteins to Mga (Hondorp et al., 2013; Valdes et al., 2018).

Although Mga is phosphorylated in the presence of glucose (Valdes et al., 2018), its expression is also impacted by CcpA (Almengor et al., 2007). In *B. anthracis*, addition of glucose increased the expression of *pagA* in an AtxA-mediated manner and it was noted that this also required a functional CcpA (Chiang et al., 2011). Conversely, Bier and colleagues reported no such alterations in *atxA* expression in the presence of glucose in the media, nor were they able to detect a difference in *atxA* expression between a WT and *ccpA* deletion strain (Bier et al., 2020). Thus, changes in the availability of glucose could alter both AtxA and Mga expression through CCR rather than through direct interaction with EI/HPr/EIIB proteins.

### The Impact of Other Sugars on PCVRs

Limited studies have comprehensively investigated whether other sugars impact the phosphorylation state of PCVRs. In *S. pyogenes*, a *ptsI* deletion mutant resulted in a strain defective in sugar uptake as well as relaying any PTS-signals to regulatory proteins (Gera et al., 2014). When this mutant was grown in chemically defined media only containing individual sugars, numerous sugars were found to be taken up exclusively through PTS-uptake pathways, and importantly, subsequent mutations to specific EIIC transporters associated with the identified sugars revealed that many of these pathways may serve redundant functions (Sundar et al., 2017). The EII complex that was most redundant in this regard was the mannose-specific EII (encoded by *manLMN*), which transported 63% of the other carbon sources tested (Sundar et al., 2017). These studies suggest that non-preferred carbon sources may play a more important role in the activation and regulatory functions of Mga than glucose in *S. pyogenes*.

Like *S. pyogenes*, *E. faecalis* encodes numerous PTS-sugar uptake pathways (Paulsen et al., 2003), and microarray analysis found that MafR differentially regulated PTS genes for fructose, lactose, and mannose (Ruiz-Cruz et al., 2015). There is also evidence that some of these sugars serve as important signals that allow *E. faecalis* to colonize epithelial surfaces and survive in the bloodstream (Zuniga et al., 2005; Vebø et al., 2009; Lindenstrauss et al., 2014). However, to date, no studies have shown that MafR can be directly phosphorylated by the PTS. Unlike AcpA, AcpB, and Mga*Spn*, each of which share conserved histidine residues within PRDs of MtlR, AtxA, and Mga, MafR appears to have no closely aligning histidine residues in putative PRDs (**Figure 2**). It is possible that for MafR, and even AcpA, AcpB, and Mga*Spn*, that the PRDs are vestigial, and these putative PCVRs have evolved to work independently of PTS input signals. Further studies must be performed in order to better understand how, if at all, the PTS directly modulates PCVRs and which carbon sources act as signals to sense host microenvironments in divergent bacterial species.

## New Advances to Study PCVR Phosphorylation

### Limitations in Current Technology

PRD phosphorylation in both PCVRs, as well as classical PRD-containing regulators, can have very different outcomes on the activity of the protein, thus complicating the understanding of how these proteins function. Phospho-mimetic/-ablative studies have proven useful in showing a cause-and-effect relationship between predicted PTS-histidine residues and protein function. Coupled with *in vivo* phosphate labeling and subsequent detection using Phos-tag™ (non-radioactive) and ^32^P-labeled (radioactive) PCVR immunoprecipitation assays have provided some insight as to whether or not PCVRs are phosphorylated inside the cell (Tsvetanova et al., 2007; Valdes et al., 2018). However, these methods have proven difficult to reproduce and don't show which amino-acid residues undergo phosphorylation *in vivo*. Phosphorylation is an important PTM that both eukaryotic and prokaryotic cells rely on for a multitude of cellular processes, and phospho-proteomics studies have proven to be essential in studying proteins that undergo phosphorylation, specifically at serine, threonine, and tyrosine residues (Ardito et al., 2017). However, the role of phospho-histidine (pHis) in nature has been severely underappreciated because it has not been possible to study these signaling pathways due to technological limitations (Kee and Muir, 2012).

### Advancements in Technology

Recent advances in mass spectrometry (MS) have shown promise for studying pHis phosphorylation in a quantitative manner. One issue with previous approaches was that any proteins containing pHis would often contain other phosphorylated residues that could result in false positives. Using a pHis-mimicking hapten (phosphoryltriazolylalanine), Jung-min and colleagues were able to develop and optimize a stable pan-pHis antibody that could enrich for proteins from a cell lysate using immunoprecipitation, and then determine the phosphorylation state with high-resolution nano-flow ultra-performance liquid chromatography-mass spectrometry (Kee et al., 2013; Kee et al., 2015). Indeed, advancements in developing pHis-recognizing antibodies are happening rapidly (Kalagiri et al., 2021). Oslund and colleagues later determined the neutral loss of phosphate from pHis-containing fragments was a result of collision-induced disassociation and used this information to develop software to predict pHis peptides, allowing them to confirm the phosphorylation of known PTS proteins and new pHis proteins in *E. coli* (Oslund et al., 2014).

Potel and colleagues have now developed a new methodology using Fe^3+^ immobilized metal affinity chromatography (IMAC) columns coupled with a more streamlined workflow to overcome the limitation of high acidity inherent in traditional IMAC chromatography (Potel et al., 2018). This allowed the ability to quantify the relative abundance between different pHis-containing proteins, thus reducing sample size and cost. New improvements in quantifying pHis immonium ions that results from cleavage during MS and by using electron-transfer-dissociation-based fragmentation has allowed detection sensitivity of pHis-containing peptides from cell lysate proteins (Potel et al., 2019). Using this approach, the pHis proteome of *E. coli* was determined and identified known (e.g., PTS components, two-component system histidine kinases) and novel pHis-containing proteins.

These advancements provide a means by which to rapidly uncover the phosphorylation state of the entire proteome, including PCVRs. Obtaining direct evidence of which histidine residues undergo phosphorylation *in vivo* would be a powerful tool to help direct subsequent phospho-mimetic/-ablative mutagenesis experiments to better understand how novel PCVRs function with respect to the PTS. pHis-LC-MS/MS would also be useful in determining which sugars and cognate EII complexes are essential in PTS-mediated PCVR signaling. Importantly, this same experimental approach could be used to simultaneously assess how specific PTS-sugars impact the regulation of PCVRs or other proteins in any bacterium of interest.

## PCVRs Share Characteristics with Nucleoid-Associated Proteins (NAPs)

### NAPs as Architectural Proteins

Both eukaryotic and prokaryotic organisms have multiple orders of chromosome organization that are mediated through the association between architectural proteins and DNA (chromatin). In eukaryotes, this organization is sustained through histones, which provide structural order to nucleic acid while undergoing PTMs that modulate gene expression (Venkatesh and Workman, 2015). Prokaryotes lack histones, yet possess analogous nucleoid-associating proteins (NAPs) that provide chromosomal order as well as transcriptional regulation (Dame et al., 2020). Despite sharing functional similarities, NAPs are believed to be evolutionarily distinct from their eukaryotic DNA-organizing counterparts and their function is a result of convergent evolution (Dame et al., 2020). Classical examples of NAPs are histone-like nucleoid structuring proteins (H-NS), structural maintenance of chromosomes proteins (SMC), factor for inversion stimulation (Fis), integration host factor (IHF), and heat-unstable protein (HU) (Hołówka and Zakrzewska-Czerwińska, 2020). A recent review by Dame et al. provides a comprehensive overview of these NAPs and their biology (Dame et al., 2020).

Despite their structural differences, NAPs share functional similarities with one another. The first is that, like histones, NAPs regulate gene expression and DNA-replication, often in response to both internal and external signals. Additionally, NAPs are highly abundant in the cytosol, spanning long regions across the entire chromosome and forming large multimeric clusters that can occupy hundreds of base-pairs at once (Li et al., 2009). NAPs like H-NS and HU also appear to mediate specific interactions with chromosome-interaction domains by recognizing the shape of DNA rather than a specific sequence (Le et al., 2013; Marbouty et al., 2015). These regions are often AT-rich and lack any NAP-specific consensus sequence (Dorman, 2014). Additionally, binding and subsequent gene activation/repression is often a function of the overall abundance of NAP oligomers that are sequestered to a specific *cis*-regulatory elements. For instance, transitioning of H-NS from a dimeric to a long oligomeric state has been shown to enhance repression of its regulated genes (Giangrossi et al., 2014).

### NAP-Like Properties of PCVRs

One of the most interesting observations that has emerged from the characterization of PCVRs is how they share many functional characteristics with NAPs. Numerous studies have found that PCVR DNA-binding is important for gene activation despite lacking sequence homology across different binding sites (McIver and Myles, 2002; Solano-Collado et al., 2012; Ruiz-Cruz et al., 2018; Toyomane et al., 2019). A recent study showed that AtxA bound to the *B. anthracis* protective antigen gene (*pagA*) promoter *in vitro* in a phosphorylation-dependent manner and, although the binding region contained stem-loop structures, there was no specific binding site identified (McCall et al., 2019). Like NAPs, promoter regions targeted by AtxA, Mga, Mga*Spn*, and MafR share low levels of homology, are AT-rich, and have intrinsic curvature (Geist et al., 1993; McIver et al., 1999; Almengor and McIver, 2004; Hadjifrangiskou and Koehler, 2008; Hause and McIver, 2012; Solano-Collado et al., 2013; Ruiz-Cruz et al., 2018; Ruiz-Cruz et al., 2019). Additionally, purified Mga and Mga*Spn* have the propensity to form insoluble oligomers *in vitro* (Hondorp et al., 2012). In the case of Mga*Spn*, this aggregation has been visualized using electron microscopy, albeit in the presence of Mga*Spn*-specific DNA fragments (Solano-Collado et al., 2012; Solano-Collado et al., 2013). Although Mga*Spn* aggregation could be a product of *in vitro* conditions that don’t accurately reflect the naturally buffered solution inside a cell, the argument that PCVR regulation is in part a function of oligomeric state is substantiated by the fact that purified Mga*Spn* and H-NS both form higher order multimers while maintaining reciprocal binding capabilities to one another’s DNA-binding regions (Solano-Collado et al., 2016).

Taken together, the observations above suggest a model that upon activation through yet discovered signaling pathways, PCVRs will undergo a conformational change impacting dimerization and DNA-binding that may be mediated through recognition of *cis*-element sequence specificity, DNA curvature, or a combination of both. Initial PCVR dimers may then serve as sites for the nucleation of long oligomers that further enhance the activation/repression of affected open-reading frames. This raises the possibility that PCVRs may exhibit dual modalities of transcriptional regulation by somehow “switching” between transcription factor and NAP modes based on the presence/absence of certain biochemical inputs. Within the Mga regulon, some *cis*-binding regions are well-conserved whereas others are highly variable. For instance, the *scpA* and *emm cis*-elements bound by Mga share a great level of sequence similarity amongst different strains of *S. pyogenes*, however other Mga-regulated loci are poorly conserved (Hause and McIver, 2012). Thus, Mga may act as a prototypical transcription factor when regulating *scpA* and *emm* genes, for example, yet control other loci that lack a consensus sequence by acting as a NAP. This will need to be investigated using genome-wide approaches that quantify occupancy of proteins on DNA such as ChIP-seq.

It is also possible that any NAP-like function of PCVRs is completely independent of oligomerization. Indeed, the EII domains of MtlR have been shown to tether MtlR to the cytoplasmic membrane *via* interaction with the mannitol permease and this mechanism is required for activity (Bouraoui et al., 2013). Similar mechanisms of spatial regulation were also described for LicT from *B. subtilis* (Rothe et al., 2013). Like these PRD-containing regulators, there are numerous NAPs like Noc and SlmA that are associated with the cell wall and remodel chromatin during cell division (Bernhardt and de Boer, 2005; Adams et al., 2015). Thus, it remains to be determined whether or not PCVRs oligomerize *in vivo* or if they function as NAPs.

## Conclusion

The PTS-pathway is a conserved system in Gram-positive bacteria that couples the import of sugars while simultaneously informing the bacteria of their respective availability and PRD-containing regulatory proteins are the major mediators of these signals. It is now clear that there is a class of proteins that can be defined as PCVRs, that exist in diverse Gram-positive pathogens and contain structural homology with classical PRD-containing regulators. Specifically, these similarities include having two N-terminal HTH-motif domains, two central PRD-like domains, and a single C-terminal EIIB-like domain. In this review, we provide a contemporary overview of the literature surrounding PCVRs, highlighting obvious structural characteristics that they share in divergent bacteria as well as potentially important functional characteristics that stem from these structures. We propose that PCVRs either evolved divergently from an ancestral PRD-containing protein or convergently due to selective pressures that drive yet defined structure-function paradigms that may have or still do relate to the function of these proteins. One possibility is that PCVRs may have acquired changes in their regulation over time, which in turn disabled their sensitivity to PTS signals, but sustained their role in regulating genes involved in metabolism and the utilization of different carbon sources. In addition to potential PTS-mediated mechanisms of action, there is a great deal of evidence suggesting that PCVRs may behave like architectural proteins. Much like NAPs, PCVRs tend to have a preference for binding DNA based on shape and flexibility rather than specific nucleotide sequences. They also appear to have the propensity to form oligomers, however, it is not clear as of now whether this is physiologically relevant in the context of transcriptional activation/repression. There is still much to be learned about this emerging class of proteins, but with novel technological advancements, we can begin to interrogate the exact mechanisms by which PCVRs operate in diverse bacterial pathogens and begin to better understand how these proteins drive virulence.

## Author Contributions

KM, JR, and MH contributed to development and writing of review. All authors contributed to the article and approved the submitted version.

## Funding

This work was supported by National Institute of Allergy and Infectious Diseases grant R01-AI047928 to KM. MH was supported in part by a NIH T32 training grant (AI089621) on Host-Pathogen Interactions.

## Conflict of Interest

The authors declare that the research was conducted in the absence of any commercial or financial relationships that could be construed as a potential conflict of interest.

## Publisher’s Note

All claims expressed in this article are solely those of the authors and do not necessarily represent those of their affiliated organizations, or those of the publisher, the editors and the reviewers. Any product that may be evaluated in this article, or claim that may be made by its manufacturer, is not guaranteed or endorsed by the publisher.
